# Human macrophage-engineered vesicles for utilization in ovarian cancer treatment

**DOI:** 10.3389/fonc.2022.1042730

**Published:** 2023-01-11

**Authors:** David Schweer, Namrata Anand, Abigail Anderson, J. Robert McCorkle, Khaga Neupane, Alexandra N. Nail, Brock Harvey, Kristen S. Hill, Frederick Ueland, Christopher Richards, Jill Kolesar

**Affiliations:** ^1^ Division of Gynecologic Oncology, Department of Obstetrics and Gynecology, College of Medicine, University of Kentucky, Lexington, KY, United States; ^2^ Markey Cancer Center, University of Kentucky, Lexington, KY, United States; ^3^ Department of Pharmacy and Practice, College of Pharmacy, University of Kentucky, Lexington, KY, United States; ^4^ Department of Chemistry, College of Arts and Sciences, University of Kentucky, Lexington, KY, United States

**Keywords:** ovarian cancer, tumor-associated macrophage (TAMs), M1 macrophage, M2 macrophage, vesicle, immunotherapy

## Abstract

**Background:**

Ovarian cancer is a deadly female malignancy with a high rate of recurrent and chemotherapy-resistant disease. Tumor-associated macrophages (TAMs) are a significant component of the tumor microenvironment and include high levels of M2-protumor macrophages that promote chemoresistance and metastatic spread. M2 macrophages can be converted to M1 anti-tumor macrophages, representing a novel therapeutic approach. Vesicles engineered from M1 macrophages (MEVs) are a novel method for converting M2 macrophages to M1 phenotype-like macrophages.

**Methods:**

Macrophages were isolated and cultured from human peripheral blood mononuclear cells. Macrophages were stimulated to M1 or M2 phenotypes utilizing LPS/IFN-γ and IL-4/IL-13, respectively. M1 MEVs were generated with nitrogen cavitation and ultracentrifugation. Co-culture of ovarian cancer cells with macrophages and M1 MEVs was followed by cytokine, PCR, and cell viability analysis. Murine macrophage cell line, RAW264.7 cells were cultured and used to generate M1 MEVs for use in ovarian cancer xenograft models.

**Results:**

M1 MEVs can effectively convert M2 macrophages to an M1-like state both in isolation and when co-cultured with ovarian cancer cells *in vitro*, resulting in a reduced ovarian cancer cell viability. Additionally, RAW264.7 M1 MEVs can localize to ovarian cancer tumor xenografts in mice.

**Conclusion:**

Human M1 MEVs can repolarize M2 macrophages to a M1 state and have anti-cancer activity against ovarian cancer cell lines. RAW264.7 M1 MEVs localize to tumor xenografts *in vivo* murine models.

## 1 Introduction

Ovarian cancer is the leading cause of death in gynecological cancers. The American Cancer Society estimates that in 2022 there will be 19,880 new cases of ovarian cancer and 12,810 deaths (1). Most patients are diagnosed at an advanced stage, with a 5-year survival rate of less than 50% (2). Patients with advanced-stage ovarian cancer are treated with combination platinum and taxane chemotherapy in the front-line setting. While many patients initially show a response to chemotherapy, the majority will ultimately relapse (2, 3). Unlike other solid tumors, immunotherapy has been largely ineffective in ovarian cancer (4, 5), emphasizing the need for novel immunotherapies to treat this disease.

Recent research suggests that tumor-supportive tumor-associated macrophages (TAMs) promote tumor vascularization and metastasis and are predominantly anti-inflammatory, M2-like macrophages (6, 7). In contrast, pro-inflammatory, M1-like macrophages can clear cancer cells and are associated with a better prognosis (8–10). A recent meta-analysis demonstrated that high numbers of TAMs are negatively associated with overall survival in multiple solid tumor types, including ovarian cancer (11). As macrophages are highly plastic, an area of growing interest is the repolarization of anti-inflammatory TAMs to pro-inflammatory TAMs as a potential mechanism of increasing the sensitivity of cancer cells to multiple therapies, including immunotherapy. Approaches to initiate macrophage repolarization include small molecule inhibitors, *in vitro*-transcribed mRNA, toll-like receptor (TLR) agonists, and siRNAs delivered *via* nanoparticles, all of which have demonstrated repolarization of M2-like TAMs to a M1 phenotype, resulting in downregulation of pro-tumor markers, such as vascular endothelial growth factor (VEGF) and transforming growth factor-beta (TGF-β), and upregulation of pro-inflammatory markers, including tumor necrosis factor-alpha (TNF-α) and interferon-γ (IFN-γ). However, the aforementioned approaches are limited because they fail to localize to tumor associated cells, and therefore heighten the potential for off-target side effects (7, 12–14). Additional approaches include increasing the antibody-dependent cell-mediated cytotoxicity (ADCC) of TAMs utilizing low-fucosylated antibodies, such as humanized glyco-engineered anti-AMHRII monoclonal antibody murlentamab, holds potential promise, *via* stimulating an antitumor adaptive immune response *via* TAM repolarization (15). Interest in using vesicles as potential therapeutics has grown significantly in recent years (16). Vesicles are structures of varying sizes that are created endogenously by cells and they can also be bioengineered by several techniques. In biological systems, vesicles enable cell-to-cell communication, *via* the transfer of proteins, lipids, and nucleic acids (17, 18). As a therapeutic modality, vesicles can encapsulate various therapeutic agents, while minimizing immunogenicity and can efficiently target the same cell type as the donor cell (16, 19, 20). This targeting property has led to the investigation of endogenous vesicles, exosomes, isolated from cancer cells to target comparable primary malignant cells (21, 22). Currently, there is limited data on the role of cancer cell exosomes to specifically target ovarian cancer. One study examined exosomes from SKOV3 ovarian cancer cells, subsequently loaded with triptolide, an antineoplastic agent, and demonstrated anti-tumor efficacy in ovarian cancer models (23). Yet, it should be noted there are significant theoretical and practical concerns with the utilization of exosomes derived from cancer cells as prior studies have suggested that tumor cell exosomes may enhance tumor progression and metastasis (17, 21, 22, 24–31).

Another approach is the utilization of vesicles derived from macrophages to target the macrophage-abundant tumor microenvironment seen in ovarian cancer (32). M1-type exosomes from RAW 264.7 cells, a murine macrophage line, can polarize unstimulated RAW 264.7 macrophages to the M1 phenotype (33). However, exosomes are limited in their therapeutic use due to low production yields and limitations in loading drug cargo. An alternative approach that has recently shown promise is bioengineering vesicles from macrophage cell membranes. These macrophages engineered vesicles (MEVs) can be formed by rupturing the cell membrane into fragments *via* nitrogen cavitation and allowing them to reconstitute into smaller distinct vesicle units. Engineered vesicles derived from the mouse RAW 264.7 cell line show similar properties as macrophage exosomes and can be loaded with a broad range of cargo, including therapeutics (34, 35).

MEVs derived from M1 macrophages can serve dual purposes; they can be used as a novel delivery vector for chemotherapeutics and can immunomodulate TAMs (35–37). Prior studies have demonstrated that mouse-derived M1 MEVs can repolarize mouse M2 macrophages back to an M1 state *in vitro* (35, 36). In addition, mouse M1 MEVs can be loaded with platinum-chemotherapeutics and have *in vitro* anti-cancer activity (36). Additional studies have shown that macrophage-derived vesicles loaded with paclitaxel have anti-cancer effects against multidrug-resistant cancer cell lines and murine breast cancer models (38, 39).

Here we describe the generation of MEVs from human peripheral blood mononuclear cells (PBMCs) that have been differentiated into macrophages. This is an advancement in our prior work by utilizing primary non-tumor human cells from fresh primary isolations (35, 36). We show that human M1 MEVs localize to both human macrophages and cancer cells and can repolarize M2 macrophages to an M1 phenotype. Human M1 MEVs display anticancer effects in co-culture with ovarian cancer cells. Additionally, using ovarian xenografts in mice, we demonstrate localization of RAW264.7 M1 MEVs to ovarian tumors *in vivo*.

## 2 Materials and methods

### 2.1 Cell lines

The ovarian adenocarcinoma cell lines: Caov-3, OVCAR3, and SKOV3 along with the murine macrophage line: RAW264.7, were obtained from ATCC. Caov-3 cells and RAW264.7 cells were maintained in Dulbecco’s Modified Eagle’s Medium (DMEM, ATCC), supplemented with 10% fetal bovine serum (FBS, Sigma). OVCAR3 cells were maintained in RPMI-1640 medium with glutamine and glucose (ATCC), supplemented with 10mg/mL insulin from bovine pancreas (Sigma) and 20% fetal bovine serum (FBS, Sigma). SKOV3 cells were maintained in McCoy’s 5a Medium Modified (ATCC), supplemented with 10% fetal bovine serum (FBS, Sigma). Cells were maintained at 37˚C with 5% CO_2_.

### 2.2 Human PBMC isolation and differentiation

Human PBMCs were isolated from buffy coats from 4-5 healthy donors (Kentucky Blood Center, Lexington, KY) by density gradient centrifugation (Ficoll-Paque Premium, GE Healthcare, Sweden) for each preparation of MEVs. Monocytes were isolated from PBMCs by immunomagnetic negative selection (EasySep Human Monocyte Enrichment Kit, Stemcell Technologies, Cambridge, MA). Human PBMC-derived monocytes were cultured in RPMI-1640 (ATCC) with 10% heat-inactivated Fetal Bovine Serum (Sigma-Aldrich, St. Louis, MO), 1% penicillin-streptomycin (Gibco), and recombinant human macrophage colony-stimulating factor (M-CSF, 50ng/mL, PeproTech, Rocky Hill, NJ) for 5-6 days. Media was replaced every 48 hours. M0 macrophages were stimulated for 24 hours with lipopolysaccharide (LPS, 20ng/mL, *In vivo*gen) plus recombinant human interferon-γ (IFN-γ, 20ng/mL, PeproTech) for M1 macrophages or with recombinant human interleukin-4 (IL-4, 20ng/mL, PeproTech) plus recombinant human interleukin-13 (IL-13, 20ng/mL, PeproTech) for M2 macrophages. Cells were maintained at 37°C with 5% CO_2_.

### 2.3 Vesicle generation and characterization

M1 MEVs were generated from human M1 macrophages using nitrogen (N_2_) cavitation. Cells were washed to remove any remaining cytokines, manually disrupted from cell flasks using a cell scraper, and then resuspended in phosphate-buffered saline (VWR) plus protease inhibitor (Thermo Scientific). N_2_ cavitation was performed by maintaining cells in a pre-chilled pressurized chamber (Parr Instruments Company, IL, USA) at 250 psi for 5 minutes at 4 ˚C. Vesicles were purified from cellular debris by centrifugation at 4 ˚C for 20 minutes at 4,000 x *g* then 10,000 x *g*. The supernatant was then withdrawn and ultracentrifuged at 100,000 x *g* for 1 hour at 4 ˚C. The subsequent pellet was washed five times with PBS and resuspended in PBS. Fluorescein-loaded human M1 MEVs were generated as described above, with the addition that the N_2_ cavitation step was performed in a 1mM solution of fluorescein in PBS. For the complete removal of free dye, a diluted vesicle suspension was subjected to an additional ultracentrifugation step at 100,000 x *g* for 60 minutes at 4°C. The mean diameter, concentration, and zeta potential values of MEVs were obtained *via* particle tracking analysis using a Zeta View PMX-120 using MEVs generated from 3.1x10^7^ human M1 macrophages. Nanoparticle tracking analysis was performed on human M1 MEVs generated from 2.8x10^7^ human M1 macrophages to determine the vesicle size distribution and concentration (NanoSight 300, Malvern Panalytical, United Kingdom).

### 2.4 Vesicle electron microscopy

The suspended sample of MEVs was fixed with 4% paraformaldehyde for 1 hour and rinsed with 1X PBS. The sample was serially dehydrated with different concentrations of ethanol from 30%, 50%, 70%, 75%, 80%, 90%, 95%, and 100% for 10 minutes. A droplet of the sample was pipetted and deposited onto a glass cover slip previously treated with 0.1% solution of poly-L-lysine^1^ to promote adhesion. Before the sample could fully dry, it was briefly immersed in ethanol (200 proof) and transferred into a critical point dryer (EM CPD 300, Leica Microsystems, Wetzlar, Germany) system. After drying, the surface of the sample was metallized by sputter coating 5 nm of platinum (EM ACE 600, Leica Microsystems, Wetzlar, Germany) to enhance surface electrical conductivity and subsequently imaged using a field-emission scanning electron microscope (SEM, Quanta 250 FEG, ThermoFisher Scientific, formerly FEI, Hillsboro, OR, USA).

SKOV3 cells were incubated with M1 vesicles for 24 hours. After incubation, the cells were washed with PBS and fixed with 4% paraformaldehyde for 40 minutes at room temperature (RT). The cells were then processed for immunogold labeled silver enhancement stain (IGSS). Cells were blocked with 3% BSA for 2 hours and then incubated with monoclonal rabbit anti-human CD86 (1:250 dilution) overnight at 4^0^C. Cells were then incubated with secondary anti-rabbit IgG Alexa Fluor^®^ 647 Fluoro Nanogold (Nanoprobes) at 1:100 dilution for 2 hours at RT. Silver enhancement was performed using HQ silver enhancement kit (Nanoprobes) for 5 minutes at RT. The cells were then washed three times with deionized water and further incubated with 0.2% osmium tetraoxide in PBS at 4C for 1 hour. Cell samples were exposed with 0.25% uranyl acetate for 1 hour at 4^0^C. Samples were then dehydrated using serial concentrations of ethanol: 50%, 70%, 90% and 100% (three times). Samples were then embedded with 100% resin. Samples were washed with resin twice, with the second wash added to samples and incubated for 45-60 minutes in a 60^0^C oven. A final resin polymerization was performed for 48 hours at 60^0^ C. Cultured cells were then separated from plates and a 100nm section was cut with a microtome and mounted on FCF-200-Cu grids. Images were acquired using a Thermo Scientific™ Talos™ F200X TEM (40).

### 2.5 Imaging of fluorescently-labeled vesicles

Fluorescein-labeled vesicles were generated as discussed previously and fixed onto a glass-bottom dish before imaging using a fluorescence microscope. Fluorescein-loaded vesicles were imaged using a 488 nm laser of 0.8 mW power and an exposure time of 200 ms.

### 2.6 Cytokine analysis

Human PBMC-derived monocytes were plated in 24-well plates at 1 x 10^6^ cells/well and cultured with M-CSF (50 ng/mL) for six days. Cells were stimulated in duplicate to M1 or M2 macrophages as previously described. M1 macrophages from the same PBMC isolation plated on separate plates were used to generate MEVs. Vesicles were washed to remove any remaining cytokines, then plated with M2 macrophages. Media supernatants were collected following a 24-hour incubation period and were assayed in duplicate using a human TNF-α Quantikine ELISA kit (R&D Systems, Inc., Minneapolis, MN). Optical density was measured using a microplate reader (Varioskan LUX, Thermo Scientific, Finland). Experiments were performed in triplicate.

### 2.7 Real-time PCR of macrophage biomarkers

Human peripheral blood monocytes were isolated, plated, and cultured for five days into differentiated M0 macrophages. M0 macrophages plated in a 6-well plate at a concentration of 5.0 x 10^5^ per well, after which macrophages were polarized to either an M1 or M2 state using LPS/IFNγ or IL4/IL13, respectively. M1 MEVs were prepared from additional M1 macrophages as previously described and were then used to treat M2 macrophages. Following an additional 24-hour incubation, RNA was purified from human macrophages (M0, M1, M2, MEV-treated M2) with RNeasy Plus Universal Mini Kit (Qiagen), and 500 ng of each sample was converted to cDNA using High-Capacity cDNA Reverse Transcription Kit (ThermoFisher Scientific) with random primers. Real-time semi-quantitative PCR measured gene expression using TaqMan Advanced Master Mix with TaqMan Gene Expression Assays (ThermoFisher Scientific). Expression of human CXCL8 (assay ID Hs00174103_m1), CXCL10 (assay ID Hs00171042_m1), relative to endogenous control GAPDH (assay ID Hs02758991_g1) was measured in triplicate using a QuantStudio 3 Real-Time PCR instrument (Applied Biosystems). Relative expression was evaluated across samples with QuantStudio Software (Applied Biosystems) using the Comparative C_T_ (ΔΔC_T_) method.

### 2.8 Co-culture of human M2 macrophages and cancer cells

For co-culture imaging experiments, human M0 macrophages were plated at 5 x 10^4^ cells/well in a 96-well clear-bottom, black-walled plate. M0 cells were stimulated to M1 or M2 for 24 hours. Caov-3 ovarian adenocarcinoma cells were then plated at 5000 cells/well with M1 or M2 macrophages. Human M1 MEVs were generated and labeled with a lipophilic dialkylcarbocyanine fluorescent dye, DiI (1,1’-Dioctadecyl-3,3,3’,3’-Tetramethylindocarbocyanine Perchlorate, Molecular Probes Inc., Invitrogen, Eugene, OR). DiI labeled vesicles were obtained by incubating MEV-resuspension with 5 µM DiI for 30 minutes at 37°C. The free dye molecules were separated from the fluorescently-labeled vesicles using a size exclusion spin column (PD MidiTrap column) following the manufacturer’s protocol. Human M1 DiI-labeled MEVs at a 10% dilution were added to Caov-3 cells, M2 macrophages, or Caov-3 plus M2 macrophage co-culture. After a 24-hour incubation period, cells were imaged at 40x with confocal microscopy (CellInsight CX7 High-Content Screening Platform). Cells were incubated with Hoescht (1:2000) for 30 minutes before imaging to label nuclei.

For cell viability experiments, human M0 macrophages were plated at 2.5-5 x 10^4^ cells/well in a 96-well plate. M0 cells were stimulated to M1 or M2 for 24 hours. Supernatant was then removed and Caov-3 ovarian adenocarcinoma cells (ATCC) were then plated at 5000 cells/well with M1 or M2 macrophages. M0, M1, and M2 macrophages and Caov-3 controls were each plated in at least duplicate. Supernatants were collected after 24 hours. A 20% or 10% dilution of human M1 MEVs was added to Caov-3 cells only and Caov-3 plus M2 cells in duplicate. Supernatants were collected after 24-hour incubation with MEVs, and wells were replaced with complete media. A cell viability assay was performed after 96 hours following the addition of MEVs according to the manufacturer’s instructions (CellTiter-Glo 2.0, Promega). Luminescence was measured with a microplate reader (Varioskan LUX). This process was repeated in the same manner with OVCAR3 cells. Experiments for both cell lines were performed in triplicate. The collected supernatants were assayed in duplicate using a human TNF-α Quantikine ELISA kit (R&D Systems, Inc., Minneapolis, MN).

### 2.9 RAW264.7 MEV generation and mouse localization experiments

RAW264.7 cells were maintained at 37°C with 5% CO_2_. RAW264.7 cells were stimulated to an M1 state using LPS/IFNγ at a concentration of 20 ng/ml for 24 hours. Cells were then manually collected using a cell scraper, and vesicles were generated in the same manner as described above. The vesicle pellet was resuspended in 2 ml of sucrose buffer (10 mM HEPES, 250 mM Sucrose pH 7.5). DiR (DiIC_18_(7); 1,1′-dioctadecyl-3,3,3′,3′-tetramethylindotricarbocyanine iodide) (ThermoFisher Scientific) was utilized as a lipophilic fluorescent dye, with 5 μl of 2 mM added to the vesicle solution and then incubated for 30 minutes at 37°C. The vesicle solution was then layered with a 50% and 10% OptiPrep™ density gradient medium. The combined solution was then ultracentrifuged at 112,000 x g for 60 minutes at 4°C. A peristaltic pump was then used to collect DiR labeled vesicles between the gradients. The collected vesicles were purified using size exclusion PD Miditrap columns (Cytiva) to remove any free dye.

Under the University of Kentucky Institutional Animal Care & Use Committee (IACUC) protocol #2017-2674, we did a transperitoneal injection of 5-week-old female BALB/c SCID mice (Jackson Lab) with 5 x 10^6^ Caov-3 cells in 100 μl of sterile PBS. After visible tumor progression, 100-200ul of labeled RAW264.7 MEVs were injected *via* lateral tail veins of *via* intraperitoneal injection in the right lower quadrant. Athymic nude homozygous 5-week-old female (Jackson Lab) were subcutaneously injected with 2.5-5.0 x 10^6^ SKOV3 cells in 100ul of sterile PBS in the dorsal shoulder region. Mice were imaged 72 hours post-injection using a LagoX Small Animal Optical Imager (Spectral Instruments) at a fluorescent excitation wavelength of 710 nm and emission of 770 nm for 10 seconds. Images were processed with Aura Imaging software (Spectral Instruments). After euthanasia, necropsy performed with tumor and organs of interest isolated and imaged independently.

## 3 Results

### 3.1 Characterization of human MEVs

**Figure 1:**
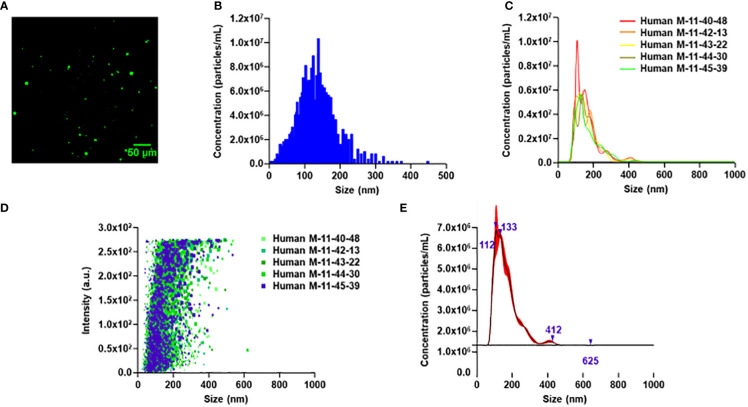
Characterization of human PBMC-derived M1 vesicles. **(A)** Wide field image of human M1 MEVs loaded with fluorescein. **(B)** Particle tracking analysis for human M1 MEVs. 2.4 x 10^7^ human M1 macrophages generated 2 x 10^10^ vesicles. **(C)** Finite track length adjustment (FTLA)/size graph obtained *via* nanoparticle tracking analysis (NTA) with five separate experimental replicates. **(D)** Intensity/size graph obtained *via* NTA with five separate experimental replicates. **(E)** Composite FTLA concentrations/size obtained *via* NTA with a mean of 165.1nm (SD: 66.4nm).

MEVs are formed *via* mechanical disruption of macrophage cell membranes with nitrogen cavitation (35). The generated cellular fragments subsequently reform into vesicles in a pressurized chamber. To determine the ability of human MEVs to encapsulate cargo, human MEVs were generated in the presence of fluorescein, a fluorescent dye. MEVs were imaged using a fluorescence microscope using a 488 nm laser of 0.8 mW power with a gain of 990 and an exposure time of 200 ms. MEVs were visible as bright punctate regions (**Figure 1A**). This illustrates that human MEVs can encapsulate cargo during vesicle generation, similar to MEVs generated from RAW 264.7 cells (35). To characterize the vesicle size distribution within an individual preparation of MEVs, we quantified the vesicle diameter and concentration using multiple particle tracking using a Zeta View PMX-120 (**Figure 1B**) and Nanosight 300 (**Figures 1C, D**). We generated vesicles from 3.1 x 10^7^ human M1 macrophages with a cavitation pressure of 250 psi, which yielded 6.6 x 10^10^ vesicles with a mean diameter of 125.1 nm (SD ± 60.2 nm). Additionally, we measured the Zeta potential at -127mV; a large negative value is an indicator of stability in an aqueous solution. Additional characterization performed with Nanosight 300 (**Figures 1C–E**) using 2.8 x 10^7^ human M1 macrophages yielding 6.45x10^11^ with a mean diameter of 165.1nm (SD ± 66.4nm).

**Figure 2:**
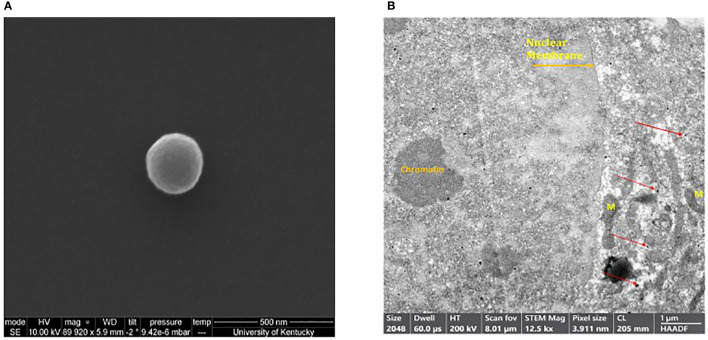
**(A)** Scanning electron microscope image of a single human M1 MEV. Sample was imaged using a field-emission scanning electron microscope. **(B)** Transmission electron microscopy of SKOV3 ovarian cell with intracellular MEVs as identified with positive silver staining for CD86. MEVs (black dots) are identified within the cell (red arrows). The nuclear membrane, chromatin, and Mitochondria (M) are also visible.

Scanning electron microscopy (SEM) was performed in order to determine the shape and morphology of the generated MEVs. MEVs were fixed and serially dehydrated prior to SEM. Examination confirmed the round smooth-edged morphology with diameter of a single MEV of~200nm (**Figure 2A**). The dense MEV spherical morphology suggests a tendency to encapsulate the cargo drug with firm stability. Utilizing transmission electron microscopy (TEM), M1 vesicles were then identified using CD86 monoclonal antibody. CD86 is a known glycoprotein found in the membrane of the antigen presenting cells, such as blood monocytes and macrophages. **Figure 2B** shows positive immunogold staining of M1 MEVs (positive for CD86) as seen as dark black silver particles within a SKOV3 cell. The SKOV3 cell membrane and nucleus containing chromatin were also visible.

### 3.2 M1 MEVs are taken up by M2 macrophages and cancer cells

**Figure 3:**
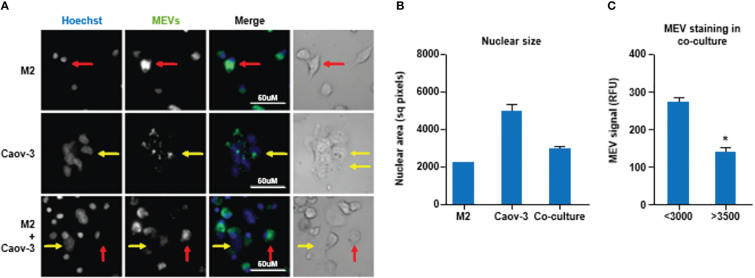
**(A)** Human macrophages display a higher uptake of human M1 MEVs compared to ovarian cancer cells. Confocal imaging of human M2 macrophages alone, Caov-3 cells alone, and co-cultured human M2 macrophages plus Caov-3 cells following a 24-hour incubation with M1 MEVs. Brightfield of co-cultured human M2 macrophages plus Caov-3 cells. Nuclei were labeled with Hoescht (1:2000, blue) and M1 MEVs were DiI-labeled (green). Representative Caov-3 cells (yellow arrows) and human macrophages (red arrows) are indicated. Scale bars indicate 50 µm. Imaging was performed at 40X magnification using a CellInsight CX7 High-Content Screening Platform. **(B)** Graph of the nuclear size mean +/- SEM showing significantly different nuclear area of the M2 cells compared to the Caov3 cells, with coculture mean between the two cell types. Greater than 100 cells were analyzed per cell type. (P<0.001 all comparisons by One-way ANOVA with Newman Keuls Multiple Comparison Test.) **(C)** MEV staining in cells with nuclei <3000 sq. pixels (M2) and >3500 sq. pixels (Caov3) from the cocultured wells only, demonstrated significantly less MEV staining in the large nuclei (Caov3) cells in the co-cultured well then the small nuclei (M2) cells as determined by unpaired two-tailed t-test (p<0.0001). * indicates a p<0.05.

Next, we examined if M1 MEVs can localize to M2 macrophages and ovarian carcinoma cells. We generated M1 MEVs labeled with DiI, a lipophilic fluorescent dye that is loaded in the membrane. MEVs were incubated with M2 macrophages, Caov-3 cells, and co-culture of M2 macrophages plus Caov-3 cells. Confocal imaging with a CellInsight CX7 High-Content Screening Platform demonstrated that both human M2 macrophages and Caov-3 cells uptake MEVs in co-culture (**Figure 3A**). Caov-3 cells and macrophages demonstrated different nuclear sizes when cocultured alone, with Caov-3 nuclei significantly larger (**Figure 3B**). While Caov-3 cells showed a low level of punctate MEVs co-localizing to the cells, most macrophages, indicated by smaller nuclei, display a distinctly higher number of MEVs (**Figure 3C**). These results show that human MEVs are capable of localizing to both human macrophages and human ovarian cancer cells *in vitro*.

### 3.3 M1 MEVs repolarize M2 macrophages

**Figure 4:**
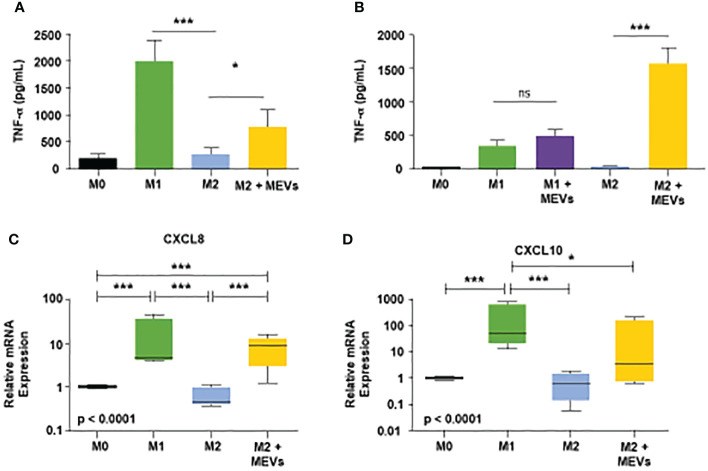
**(A)** Human M1 vesicles repolarize M2 macrophages. Human PBMC-derived monocytes were cultured with M-CSF (50ng/mL) for six days. Cells were stimulated, and supernatants were assayed for human TNF-α after 24 hours. From left to right on the graph: M0 macrophages (black), M1 cells polarized with LPS plus IFN-γ (20ng/mL each, green, M2 polarized with IL-4 plus IL-13 (20ng/mL each, blue, and M2 cells treated with M1 vesicles (yellow). Statistical analysis performed with Statistical analysis performed with one-way ANOVA with *post-hoc* Tukey’s Multiple Comparison Test (*p < 0.05; ***p < 0.001). Error bars indicate SD. **(B)** Human PBMC-derived monocytes were cultured, plated, and stimulated to respective states as described above. After 24 hours, supernatant was removed and M1 vesicles were added to M1 and M2 cells. After an additional 24 hours supernatants were collected and subsequently assayed for human TNF-α. Statistical analysis performed with one-way ANOVA with *post-hoc* Tukey’s Multiple Comparison Test (*p < 0.05; ***p < 0.001). Error bars indicate SD. **(C, D)** CXCL8 and CXCL10 mRNA expression as biomarkers of human macrophage polarization. Total RNA was purified from human M0, M1, M2 macrophages, and M2 macrophages treated with M1 MEVs (M2 + MEVs) and analyzed by real-time PCR. Relative expression (versus M0 macrophages) of CXCL8 and CXCL10 was measured in 4 independent experiments and summarized in box and whisker plots (median, interquartile range, 5th-95th percentile). Statistical analyses were performed with Kruskal-Wallis tests followed by Dunn’s Multiple Comparison tests (*p < 0.05; ***p < 0.001). ns, not significant.

Next, we tested if human M1 MEVs can repolarize M2 macrophages to an M1-like, pro-inflammatory phenotype. We compared the production of the pro-inflammatory cytokine TNF-α in M1 macrophages, M2 macrophages, and M2 macrophages incubated with M1 MEVs. We observed high levels of TNF-α, measured *via* ELISA, in the M1 macrophages and significantly lower TNF-α in the M2 culture and in controls (Mean ± SD pg/ml: M1 vs. M2: 2021 ± 383.8 vs. 259.9 ± 133.7, p<0.001, M1 MEVs+M2 vs. M2: 787.5 ± 298.3 vs. 259.9 ± 133.7 p<0.05) (**Figure 4A**). In contrast, we observed an increase in TNF-α in M2 macrophages that were incubated with M1 MEVs, indicating that M1 MEVs can repolarize M2 macrophages towards a pro-inflammatory, M1-like macrophage phenotype. **Figure 4B** demonstrates the difference in TNF- α levels of M1+M1 MEVs vs M1 cells alone is not significant. However, M2+ M1 MEVs vs M2 cells is statistically significant. From this data we’ve concluded that the MEVs alone are not the sole driver of the experimental increased TNF-α levels, but rather the interaction with the M2 cells *via* repolarization. The comparatively lower TNF-α levels seen in the M1 between **Figures 4A, B** is likely secondary to the difference in analyzed time points (24 vs 48 hrs) and experimental methodology. We subsequently sought to validate M1 MEV repolarization of M2 macrophages *via* real-time PCR of mRNA expression of CXCL8 and CXCL10 proteins. **Figure 4C** shows significant differences in the relative expression of CXCL8 in M2 cells alone compared to M2 cells treated with M1 MEVs (p<0.0001). This finding was not demonstrated in relative mRNA expression of CXCL10 (**Figure 4D**). CXCL8 expression is marker for M1 macrophages (41–43). Therefore, based on CXCL8 mRNA expression, M1 MEVs can repolarize M2 to an M1 state. Taken together, M1 MEVs can repolarize M2 macrophages into an M1-like phenotype based on both cytokine secretion and mRNA expression profiles.

### 3.4 Human M1 MEVs repolarize M2 macrophages in co-culture

**Figure 5:**
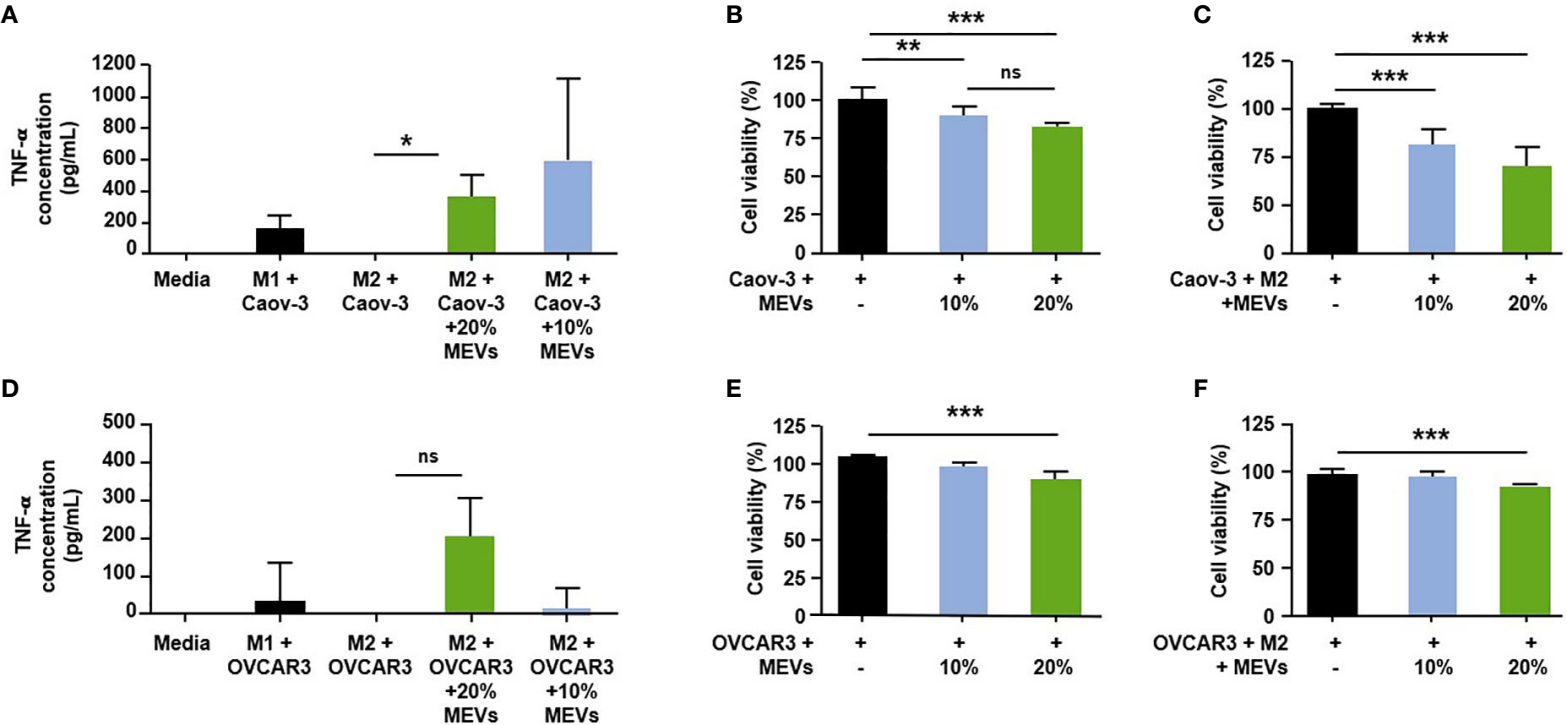
Human M1 MEVs shift co-cultured M2 macrophages to M1 phenotype. **(A, D)**. Supernatants were collected 24 hours after the addition of M1 MEVs (48 hours after macrophage plating). Supernatants were assayed in duplicate using a human TNF-α Quantikine ELISA kit (R&D Systems, Inc.). Significance was assessed with a two-tailed paired t-test **(A, D)** ; p = 0.0259 & p=0.18, respectively). Human M1 MEVs show dose-dependent inhibition of cell viability in co-cultured cells. Graphs indicate the percent cell viability of the **(B)** Caov-3 cancer cells alone and **(E)** OVCAR3 cancer cells alone treated with M1 MEVs or **(C)** Caov-3 and **(F)** OVCAR3 co-cultured cancer cells plus M2 macrophages treated with M1 MEVs. Cell viability was measured at 96 hours (CellTiter-Glo 2.0). % of MEVs refers to the relative percentage of supernatant with MEVs added. The percent cell viability was calculated by comparing cells treated with M1 MEVs to the respective untreated control. Statistical analyses were performed using Kruskal-Wallis with Dunn’s Multiple Comparison posthoc test (*p < 0.05; **p < 0.01; ***p < 0.001). Experiments were performed in triplicate. Bars correspond to SEM. * indicates a p<0.05.

To test if M1 MEVs can convert M2 TAMs to a pro-inflammatory phenotype, we cultured human M2 macrophages with the Caov-3 or OVCAR3 ovarian cancer cell lines and treated the co-cultured cells with M1 MEVs. Co-cultured cells treated with M1 MEVs show an increase in the pro-inflammatory cytokine, TNF-α (Mean ± SD pg/ml: M2+Caov-3+M1 MEVs vs M2+Caov-3; 383.6 ± 120.4 vs. 0.1389 ± 20.03, p<0.05, M2+OVCAR3+M1 MEVs vs M2+OVCAR3: 207.1 ± 170.2 vs -45.65 ± 55.35 p=0.18) (**Figures 5A, D**), suggesting that M1 MEVs convert M2 TAMs to an M1 phenotype. The comparatively lower TNF-α levels seen in the M1 plus cancer cells (**Figures 5A, D**) compared to the high levels of TNF-α in the M1 macrophages alone (**Figure 4**) is likely secondary to the difference in time points (24 vs 48 hrs) and experimental methodology.

We then tested if M1 MEVs are capable of inhibiting cell viability. M1 MEVs at high concentrations has an inhibitory effect in both Caov-3 (Mean ± SD 100.0 ± 8.232 vs 82.27± 2.853, p<0.0001) and OVCAR3 cell lines (Mean ± SD 100.0 ± 5.710 vs 87.69± 11.62, p<0.05) (**Figures 5B, E**), with continued significant decreases appreciated at a lower dose (10%) in Caov-3 (Mean ± SD: 100.0 ± 8.232 vs 87.95± 6.069, p<0.0001). Interestingly, in Caov-3 this inhibition appears to be dose-dependent and is significantly higher in the co-cultured cells as compared to cancer cells alone (Mean ± SD 100.0 ± 2.930 vs. 70.54 ± 9.955, p<0.0001) (**Figure 5C**), indicating that MEVs are more effective in the presence of pro-inflammatory macrophages. The inhibition seen in OVCAR3 cells co-cultured with M2 macrophages is more modest but still significant at a high MEV dose (Mean ± SD 100.0± 6.821 vs 93.61 ± 5.558, p < 0.01) (**Figure 5F**).

### 3.5 RAW264.7-derived M1 MEVs localize to ovarian xenografts *in vivo*

**Figure 6:**
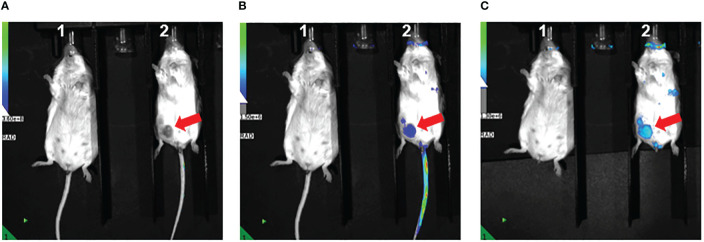
RAW.264.7 M1 polarized vesicles localize to Caov-3 tumor xenografts *in vivo*. **(A)** Two BALB/c-SCID mice displayed – one without tumor (left) and one with visible tumor (right) marked by the arrow. The mouse on the right was injected with 100 µl of fluorescent dye-labeled vesicles and imaged 72 hours post-injection. The fluorescent overlay was reduced to display visible tumor for comparison **(B)** Same mice shown in A with clear fluorescent uptake seen in the vicinity of the tumor in the right lower quadrant displayed in Image **(A, C)** Tail veins covered to reduce emission background, displaying accentuated M1 MEV localization to the tumor.

**Figure 7:**
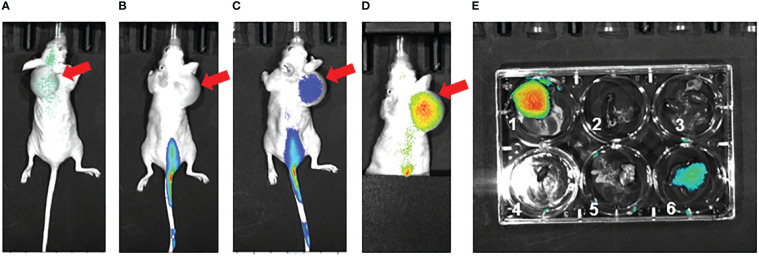
RAW.264.7 M1 polarized vesicles localize to SKOV3 tumor xenografts *in vivo via* an intravenous route. **(A)** Preinjection (0 hr – immediately prior to injection) of single athymic nude mouse with SKOV3 tumor xenograft in dorsal shoulder region (red arrow). The mouse was injected with 100 µl of fluorescent dye-labeled vesicles and imaged **(B)** 24 hours post-injection and **(C)** 72 hours post-injection. There is clear fluorescent uptake seen in the tumor. **(D)** 72 hours post-injection Tail vein covered to reduce emission background, displaying accentuated vesicle localization to the tumor 72 hours post injection. **(E)** Post-necropsy with 1=tumor, 2=spleen, 3=kidneys, 4=heart, 5=lungs, 6=liver; there is accentuated localization to the tumor. The size of the subcutaneous lesion resulted sagittal instability and displacement to the right over the time series.

**Figure 8:**
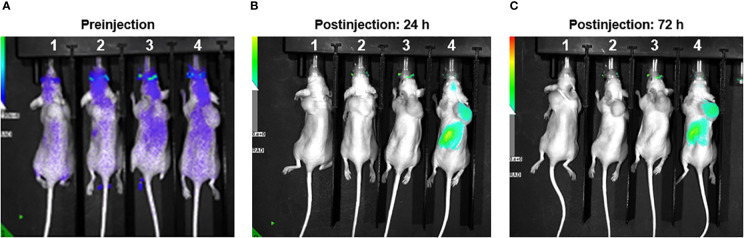
RAW.264.7 M1 polarized vesicles localize to SKOV3 tumor xenografts *in vivo via* an intraperitoneal route. **(A)** Four athymic nude mice displayed – each with SKOV3 tumor xenograft in the dorsal shoulder region. Image taken preinjection for comparison purposes. **(B)** Mice 1 & 2 injected with 200ul of sterile PBS. The two mice on the right were injected with 50ul (Mouse 3) and 100ul (Mouse 4) of DiR labeled vesicles and imaged 24 hours post-injection. There is clear localization of fluorescent uptake in the vicinity of the tumor in the far-right mouse. The fluorescent overlay was reduced to display visible tumor for comparison. **(C)** Same mice shown with clear and persistent fluorescent uptake seen in far-right mouse’s tumor.

As part of a pilot experiment, we sought to demonstrate the localization of M1 MEVs to human tumor xenografts. A BALB/c SCID mouse was injected transperitoneally with Caov-3 ovarian cancer cells and developed a visible tumor xenograft in the abdominal right lower quadrant approximately seven months post-injection. Fluorescent DiR-labeled M1 MEVs were created from RAW264.7 cells and were injected *via* lateral tail vein. Importantly, RAW164.7 are a mouse macrophages cell line. The mouse was imaged 72hrs post-injection (**Figure 6**) using appropriate corresponding emission and excitation wavelengths for DiR. An additional mouse (left) without a tumor xenograft was not injected was imaged for baseline null comparison purposes. The dye-labeled MEVs demonstrate precise localization to the tumor (**Figures 6B, C**). Additional pilot experiments were performed with athymic nude mice injected subcutaneously with SKOV3 ovarian cancer cells xenografts in the mouse scapular region. Fluorescent DiR-labeled M1 MEVs were created from RAW264.7 cells and were injected *via* lateral tail vein (**Figure 7**) or intraperitoneally (**Figure 8**). Post-necropsy images demonstrate localization of M1 MEVs to tumor (**Figure 7E**). Intermittent fluorescent signalling demonstrated in the murine cranium at 24 hours is noted, but desists at 72hours. This is suggestive of a transient circulatory phenomenon or may reflect additional M2 macrophage target populations.

## 4 Discussion

While there have been several recent advances in immunotherapy for other gynecological malignancies [cervical (25) and uterine (26)], success in ovarian cancer has been limited (27). This lack of activity in ovarian cancer is thought to be related to infiltration of TAMs, which render cancer “cold” and thus immunotherapy ineffective (34, 44). Therefore, strategies to repolarize M2 macrophages to the M1 phenotype may promote anti-cancer activity. Our study, the first to use MEVs derived from human blood monocytes, effectively demonstrates that M1 MEVs can localize primarily to M2 macrophages when co-cultured with ovarian cancer cells and treatment with M1 MEVs repolarizes M2 macrophages to an anti-tumor M1 state with subsequent anti-cancer activity. This effect was demonstrated both in cancer cells alone and with macrophages co-cultured in the presence of cancer cells. Since ovarian cancer cells themselves are significant drivers for macrophage polarization to an M2 state (45), repolarization within co-culture is particularly salient as it suggests the capacity of MEVs to overcome an innate preferential differentiation towards the protumor M2 state.

Macrophages are the most abundant immune system cells within the tumor microenvironment and compose up to 50% of a tumor’s volume (46–48). A major benefit of exosome formulations from macrophages is the inherent targeting properties exhibited by their origin cell (18). Exosomes derived from human cells are non-immunogenic compared to liposomal formulation (18). Therefore, the use of exosome-like MEVs derived from human blood cells has the potential to avoid off-target immunogenic effects while honing in on macrophage-laden tissue (e.g., tumors). Additionally, engineered macrophage vesicles carry a higher yield potential than other endogenous sources while avoiding a cancer-derived source that could impact tumorigenesis (27, 49, 50).

One of the main strengths of this study is the exclusive use of non-carcinoma human-derived cells. This eliminates any future translational risk of reintroducing tumor-derived cells into the patient. Another major strength is the immunological and therapeutic potential of M1 MEVs that is demonstrated using several ovarian cancer cell lines. Caov-3 and OVCAR3 are both BRCA wild-type, however, Caov-3 is platinum-sensitive whereas OVCAR3 are platinum resistant. In murine models, SKOV3 is an aggressive platinum resistant cell line that displays rapid xenograft growth. Additionally, pilot animal data demonstrate precise localization of dye-labeled mouse M1 MEVs to ovarian cancer tumor xenografts in mice. This is an intriguing finding and provides further evidence for the tumor precision of MEVs. Localization was seen in both intravenous and intraperitoneal administration routes. This is of compelling interest as ovarian cancer is a peritoneal disease and intraperitoneal chemotherapy has a long-studied role in the treatment of the disease (51, 52). Limitations include a lack of *in vivo* modeling to demonstrate sustained macrophage repolarization. In terms of generalization of *in vivo* models, SCID and nude mice are particularly immunosuppressed, future modeling using syngeic murine models may more accurately reflect physiologic conditions and reveal the interplay of circulating MEVs with the immune system targets. Additionally, there was high variability and size heterogeneity seen with the vesicle preparation that may be ameliorated in future studies with further filtration methods. Additional characterization methods of the vesicles *via* transmission electron microscopy is warranted. While promising as a therapeutic avenue, significant obstacles remain prior to transition from a preclinical to clinical approach, including standardization of MEV characterization, dosing, precision of imaging localization, and delineation of off-target effects. Future research will be needed to evaluate the role of drug-loaded MEVs as another therapeutic approach and evaluate *in vivo* efficacy in terms of distribution, toxicity, and tumor response.

## 5 Conclusions

The studies described are the first to demonstrate that human-derived M1 MEVs can serve as immunomodulatory agents by repolarizing M2 macrophages to an M1-like state. This effect was seen in M2 macrophages when cultured alone and in co-culture with ovarian cancer cells. Overall, human-derived M1 MEVs effectively repolarize M2 macrophages. Initial pilot data demonstrates that M1 MEVs target ovarian tumor xenografts. Future *in vivo* studies are warranted.

## Data availability statement

The original contributions presented in the study are included in the article/supplementary material. Further inquiries can be directed to the corresponding author.

## Ethics statement

The animal study was reviewed and approved by University of Kentucky Institutional Animal Care & Use Committee (IACUC).

## Author contributions

Conceptualization, AA, DS, CR, and JK; methodology, DS, AA, AN, KN, and NA; validation, DS, AA, NA, AN, KN, BH, and JRM; formal analysis, DS, AA, AN, NA, JRM, KN, KH, and BH; data curation, AA and DS; writing—original draft preparation, AA and DS; writing—review and editing, DS, AA, JRM, KH, CR, FU, BH, KN, AN, and JK; visualization, AA, KH, DS, and KN; supervision, JK; funding acquisition, DS and JK. All authors have read and agreed to the published version of the manuscript.
